# Risk Factors for Hydroxychloroquine Retinopathy and Its Subtypes

**DOI:** 10.1001/jamanetworkopen.2024.10677

**Published:** 2024-05-09

**Authors:** April M. Jorge, Ronald B. Melles, Michael F. Marmor, Baijun Zhou, Yuqing Zhang, Hyon K. Choi

**Affiliations:** 1 Division of Rheumatology, Allergy, and Immunology, Massachusetts General Hospital, Boston; 2 Harvard Medical School, Boston, Massachusetts; 3 Department of Ophthalmology, Kaiser Permanente Northern California, Oakland; 4 Department of Ophthalmology, Stanford University School of Medicine, Stanford, California

## Abstract

**Importance:**

The major toxic effect of hydroxychloroquine is retinopathy. Thus, current guidelines recommend limiting the dose and screening annually for retinopathy among all long-term users, but individual patient factors may be associated with retinopathy risk.

**Objective:**

To identify risk factors beyond hydroxychloroquine dose and duration of use for hydroxychloroquine retinopathy.

**Design, Setting, and Participants:**

This cohort study of 4677 patients in the Kaiser Permanente Northern California integrated health network who initiated hydroxychloroquine, continued treatment, and underwent retinopathy screening after 5 years of use was conducted from July 1, 1997, to December 31, 2020, with up to 15 years of follow-up. Statistical analysis was performed in August 2023.

**Exposure:**

Candidate risk factors included age at hydroxychloroquine initiation, sex, race and ethnicity, indications, chronic kidney disease (CKD), liver disease, diabetes, tamoxifen use, and medications that interact with hydroxychloroquine metabolism. Hydroxychloroquine dose was assessed from pharmacy dispensing records.

**Main Outcome and Measures:**

Incident hydroxychloroquine retinopathy was adjudicated from masked review of guideline-recommended screening studies and classified as parafoveal or pericentral pattern. Multivariable Cox proportional hazards regression was used to assess potential risk factors for hydroxychloroquine retinopathy within 15 years of initiation.

**Results:**

Of 4677 long-term hydroxychloroquine users (mean [SD] age at initiation, 52.4 [14.1] years; 3877 women [82.9%]), 125 patients developed hydroxychloroquine retinopathy within 15 years (102 parafoveal, 23 pericentral). Older age at time of hydroxychloroquine initiation was associated with retinopathy risk, with adjusted hazard ratios (HRs) of 2.48 (95% CI, 1.28-4.78) for those aged 45 to 54 years, 3.82 (95% CI, 2.05-7.14) for those aged 55 to 64 years, and 5.68 (95% CI, 2.99-10.79) for those aged 65 years or older compared with those younger than 45 years. The risk of retinopathy was higher among females than males (HR, 3.83 [95% CI, 1.86-7.89]), among patients with CKD stage 3 or greater (HR, 1.95 [95% CI, 1.25-3.04]), and among individuals with tamoxifen use (HR, 3.43 [95% CI, 1.08-10.89]). The likelihood of pericentral retinopathy was higher among Asian patients (HR, 15.02 [95% CI, 4.82-46.87]) and Black patients (HR, 5.51 [95% CI, 1.22-24.97]) compared with non-Hispanic White patients.

**Conclusions and Relevance:**

This study suggests that increasing age, female sex, CKD stage 3 or greater, and tamoxifen use were associated with a higher risk of hydroxychloroquine retinopathy, whereas being younger than 45 years at hydroxychloroquine initiation and male sex were associated with a lower risk. Race and ethnicity were also associated with the pattern of retinopathy. These factors should be incorporated into hydroxychloroquine dosing decisions.

## Key Points

**Question:**

What factors beyond hydroxychloroquine dose and duration of use are associated with the risk of hydroxychloroquine retinopathy?

**Findings:**

In this cohort study of 4677 long-term hydroxychloroquine users with masked adjudication of retinopathy outcomes, increasing age, female sex, chronic kidney disease stage 3 or greater, and tamoxifen use were each associated with a higher risk of hydroxychloroquine retinopathy.

**Meaning:**

This study suggests that these factors should be considered when making decisions about hydroxychloroquine dosing and screening for hydroxychloroquine retinopathy.

## Teaser

This cohort study investigates demographic and clinical patient characteristics associated with risk of hydroxychloroquine retinopathy.

## Introduction

Hydroxychloroquine is an important treatment for rheumatic diseases because of its relative safety, tolerability, and low cost profile. It remains a mainstay of treatment for patients with systemic lupus erythematosus (SLE), with benefits including improved survival and prevention of SLE flares and damage.^1,2,3^ However, its major adverse event is hydroxychloroquine retinopathy, an ocular toxic effect that can develop over long-term use. Hydroxychloroquine retinal toxic effects are characterized by thinning of the outer retina and eventual damage of the retina pigment epithelium and can lead to loss of vision in advanced stages.^4^ With typical dose ranges used in the treatment of rheumatic and dermatologic conditions, the risk of hydroxychloroquine retinopathy is rare within the first 5 years of use, but the overall risk of retinopathy increases to 8.6% after 15 years of use.^5^

Hydroxychloroquine daily dose and duration of use are major established risk factors for hydroxychloroquine retinopathy; prior studies have found long-term use of hydroxychloroquine with dosing over 5 mg/kg/d to be associated with higher long-term risks of hydroxychloroquine retinopathy, and the risk of retinopathy increases markedly after 10 years of use.^5,6^ The potential association of other risk factors with hydroxychloroquine retinopathy is less clear. Prior studies of such risk factors have been limited by small sample size, prevalent case analyses, and potential misclassification of outcomes.^6,7,8,9,10,11,12^ Furthermore, while the classic description of hydroxychloroquine toxic effects from studies of primarily White populations in Europe and the US has been a parafoveal retinopathy pattern, a pericentral retinopathy pattern has now been recognized as the dominant pattern among Asian populations.^10,13^ It is unknown whether other risk factors vary according to the pattern of hydroxychloroquine retinopathy. To inform the optimal use of this medication, we assessed the risk factors for incident hydroxychloroquine retinopathy within a large-scale, longitudinal cohort of patients who initiated hydroxychloroquine and continued the medication for at least 3 years.

## Methods

### Study Setting and Population

All patients 18 years of age or older who were enrolled in the US integrated health network Kaiser Permanente Northern California (KPNC) and initiated hydroxychloroquine between July 1, 1997, and December 31, 2014, were identified, and patients were followed up through December 31, 2020, for up to 15 years of use. We excluded patients with fewer than 5 years of continuous enrollment in KPNC from hydroxychloroquine initiation and required at least 1 prescription for hydroxychloroquine after 5 years (ie, 60 months), as previously described,^5^ to identify a cohort of patients at risk for hydroxychloroquine retinopathy. The KPNC database captures demographic characteristics, biometrics, medications, longitudinal primary and specialty care, laboratory test data, and imaging studies, including digital images for all spectral domain–optical coherence tomography studies that were obtained in routine hydroxychloroquine retinopathy screening. The KPNC database also includes comprehensive pharmacy records, including near-complete capture of prescription drug dispensation over this period. This study was approved by the KPNC institutional review board and the Mass General Brigham institutional review board, and informed consent was waived because the study involved no more than minimal risk. This study followed the Strengthening the Reporting of Observational Studies in Epidemiology (STROBE) reporting guideline.

### Study Design

We conducted a longitudinal cohort study to assess risk factors for incident hydroxychloroquine retinopathy and its subtypes. Patients were followed up from hydroxychloroquine initiation until the date of their last hydroxychloroquine retinopathy screening study. Because recommended retinopathy screening begins at 5 years of use (ie, 60 months after hydroxychloroquine initiation), this was the baseline time point for the risk factors analyses.

### Outcome

We assessed the outcome of hydroxychloroquine retinopathy with masked readings of spectral domain–optical coherence tomography scans and adjudication by 2 expert ophthalmologists (R.B.M. and M.F.M.), as previously described.^5^ We considered the dates of earliest abnormal scans as the dates of retinopathy onset. Each retinopathy case was classified as parafoveal or pericentral pattern according to the primary location of retinal damage. The parafoveal pattern was defined as damage to photoreceptors and/or retina pigment epithelium in a ring between 2° to 6° from the center of the fovea, and the pericentral pattern was defined as corresponding damage located 8° or more from the center of the fovea.^13^

### Candidate Risk Factors

Potential risk factors were selected based on prior knowledge and/or possible biological roles in the susceptibility to hydroxychloroquine retinopathy.^4^ Risk factors were assessed as of 5 years of hydroxychloroquine use, the recommended time to begin annual hydroxychloroquine retinopathy screening. Hydroxychloroquine is estimated to undergo 25% to 40% renal clearance,^14^ and chronic kidney disease (CKD) has been associated with an increased risk of hydroxychloroquine retinopathy in prior studies.^6^ Because the association between the degree of kidney dysfunction and the risk of retinopathy was not well established, we assessed the association between continuous estimated glomerular filtration rate (eGFR) and hydroxychloroquine retinopathy. We also assessed the candidate risk factor of CKD stage 3 or greater, defined as annual mean eGFR less than 60 mL/min/1.73 m^2^. Hydroxychloroquine also undergoes metabolism in the liver by cytochrome P450 (CYP) enzymes, primarily including CYP2D6, CYP2C8, or CYP3A4,^15^ so use of 1 or more medications that inhibit one of these CYP enzymes was evaluated as a candidate risk factor for hydroxychloroquine retinopathy (eTable in Supplement 1).^15,16^ We also assessed liver disease, which has been proposed as a possible risk factor.^17^ Tamoxifen, a medication used to treat hormone-sensitive breast cancer, which can itself cause retinopathy, was considered another potential risk factor because 1 prior study found tamoxifen use to be associated with increased risk of hydroxychloroquine retinopathy.^6^

We included other purported risk factors that may be associated with hydroxychloroquine metabolism or exposure,^4^ including age at hydroxychloroquine initiation, sex, race and ethnicity, and indication for hydroxychloroquine use (eg, SLE or other connective tissue diseases, rheumatoid arthritis or other inflammatory arthritis, or other indications, which primarily included dermatologic conditions).^7,12,18^ Race and ethnicity were assessed due to prior studies having found an association between Asian race and the pericentral retinopathy pattern.^10^ Race and ethnicity were ascertained from the KPNC enrollment database where they are recorded as mutually exclusive categories of Asian, Black, Hispanic, non-Hispanic White, and unknown. As diabetes may be associated with a separate retinopathy, we also assessed for type 2 diabetes as a potential risk factor for hydroxychloroquine retinopathy (defined by a mean annual hemoglobin A_1c_ level >6.5% [to convert to proportion of total hemoglobin, multiply by 0.01]).

Because hydroxychloroquine exposure is necessary for the development of hydroxychloroquine retinopathy and the weight-based dose is an established risk factor for hydroxychloroquine retinopathy, we assessed the hydroxychloroquine weight-based dose in milligrams per kilogram per day from pharmacy dispensing records. We categorized the dose as less than or equal to 5, 5 to 6, or greater than 6 mg/kg/d; dosing category was defined by least 80% of days covered by a given dose range in the fifth year of hydroxychloroquine use (ie, months 48-60 after hydroxychloroquine initiation). We also assessed the cumulative hydroxychloroquine dose in the first 5 years of use.

Demographic variables, comorbidities, and medication records were available for all patients. For 3 patients with missing creatinine levels, we imputed the mean value of 0.90 mL/min/1.73 m,^2^ classified as no CKD. For 2 female patients with missing body weight, we imputed the sex-specific mean of 75.6 kg.

### Statistical Analysis

Statistical analysis was performed in August 2023. We examined the association between candidate risk factors and the risk of hydroxychloroquine retinopathy using Cox proportional hazards regression models to determine the hazard ratios (HRs) and 95% CIs and the cumulative incidence up to 15 years of hydroxychloroquine use. As we sought to evaluate the association of risk factors beyond the established risk factor of hydroxychloroquine dose, the adjusted models included weight-based hydroxychloroquine dose and cumulative dose in the first 5 years of use in each risk factor analysis. The adjusted models also included age, sex, and race and ethnicity, which may be associated with the development of other risk factors. The indication for hydroxychloroquine use and CKD status may be associated with the hydroxychloroquine dose and retinopathy risk, so we adjusted for age, sex, race and ethnicity, indication for hydroxychloroquine use, and CKD in the risk factor analysis for hydroxychloroquine weight-based dose. We repeated this risk factor analysis for the outcomes of parafoveal or pericentral retinopathy. We tested the proportional hazards assumption for each model and found no violations of the assumption.

To characterize the association between kidney function and the risk of hydroxychloroquine retinopathy, we used restricted cubic splines to generate a smoothed eGFR-HR curve. The reference eGFR was 90 mL/min/1.73 m^2^ or greater, with knots at 45, 60, and 90 mL/min/1.73 m^2^. To characterize the dose-response trend between hydroxychloroquine weight-based dose at 5 years of hydroxychloroquine use and the risk of hydroxychloroquine retinopathy, we used restricted cubic splines to generate a smoothed dose-HR curve. The reference weight-based dose was 5 mg/kg/d, with knots at 4, 5, and 6 mg/kg/d. All *P* values were 2-sided with a significance threshold of *P* < .05, and the analyses were conducted using SAS, version 9.4 (SAS Institute Inc).

## Results

### Patient Demographics

Of 4677 long-term hydroxychloroquine users, 3877 [82.9%] were female, 800 [17.1%] were male, and the mean [SD] age at hydroxychloroquine initiation was 52.4 (14.1) years (Table 1). Most patients were non-Hispanic White (2716 [58.1%]), while 642 (13.7%) were Asian, 491 (10.5%) were Black, and 828 (17.7%) were Hispanic. After 5 years of use, 537 patients (11.5%) had CKD stage 3 or greater. Less than 1% of patients (17 [0.4%]) concurrently used tamoxifen. Although many patients (2858 [61.1%]) initiated a hydroxychloroquine dose over 5 mg/kg/d, the mean (SD) initial weight-based dose was 4.4 (1.5) mg/kg/d. By 5 years of use, only 1608 (34.4%) were using a hydroxychloroquine dose over 5 mg/kg/d, with a mean (SD) dose of 3.2 (1.9) mg/kg/d.

**Table 1.** Characteristics of the Study Cohort at Hydroxychloroquine Initiation and 5 Years of Use^a^

Characteristic	At hydroxychloroquine initiation (N = 4677)	At 5 y of use (N = 4677)
Age, mean (SD), y	52.4 (14.1)	57.4 (14.1)
Sex, No. (%)		
Female	3877 (82.9)	3877 (82.9)
Male	800 (17.1)	800 (17.1)
Race and ethnicity, No. (%)		
Asian	642 (13.7)	642 (13.7)
Black	491 (10.5)	491 (10.5)
Hispanic	828 (17.7)	828 (17.7)
Non-Hispanic White	2716 (58.1)	2716 (58.1)
Indication for hydroxychloroquine, No. (%)		
Dermatologic conditions or other	383 (8.2)	383 (8.2)
Rheumatoid arthritis or other inflammatory arthritis	2690 (57.5)	2690 (57.5)
SLE or other connective tissue diseases	1604 (34.3)	1604 (34.3)
Weight, mean (SD), kg	78.1 (20.3)	78.0 (20.7)
Estimated GFR, mean (SD), mL/min/1.73 m^2^	89.5 (20.5)	86.5 (20.5)
CKD stage ≥3, No. (%)	407 (8.7)	537 (11.5)
Diabetes, No. (%)	260 (5.6)	324 (6.9)
Liver disease, No. (%)	110 (2.4)	105 (2.3)
Medication use, No. (%)		
CYP2D6, CYP2C8, or CYP3A4 inhibitors	515 (11.0)	872 (18.6)
Tamoxifen	17 (0.4)	18 (0.4)
Weight-based hydroxychloroquine dose, >5 mg/kg, No. (%)	2858 (61.1)	1608 (34.4)
Weight-based hydroxychloroquine dose, mean (SD), mg/kg	4.4 (1.5)	3.2 (1.9)
Cumulative hydroxychloroquine dose, mean (SD), g	NA	454.5 (194.6)

Abbreviations: CKD, chronic kidney disease; CYP, cytochrome P450; GFR, glomerular filtration rate; NA, not applicable; SLE, systemic lupus erythematosus.

^a^ The 5 years of use characteristics were assessed between 48 and 60 months after hydroxychloroquine initiation.

### Risk Factors for Hydroxychloroquine Retinopathy

Overall, 125 patients developed incident hydroxychloroquine retinopathy within 15 years of use, with a parafoveal pattern among 102 patients and pericentral pattern among 23 patients. The mean (SD) duration between the last normal screening and the first abnormal hydroxychloroquine screening study was 1.6 (1.2) years. The risk of retinopathy was associated with older age at the time of hydroxychloroquine initiation (Table 2). Compared with being younger than 45 years, multivariable adjusted HRs increased to 2.48 (95% CI, 1.28-4.78) for those aged 45 to 54 years, 3.82 (95% CI, 2.05-7.14) for those aged 55 to 64 years, and 5.68 (95% CI, 2.99-10.79) for those aged 65 years or older. Female sex was also associated with a higher risk of retinopathy compared with males (adjusted HR, 3.83 [95% CI, 1.86-7.89]), who had a less than 3% cumulative incidence of hydroxychloroquine retinopathy through 15 years of use. Older age and female sex were also associated with the risk of parafoveal retinopathy pattern but were not significantly associated with pericentral retinopathy (Table 3).

**Table 2.** Risk Factors for Hydroxychloroquine Retinopathy

Characteristic	Patients, No.	Events, No.	15-y Cumulative incidence, % (95% CI)	Hazard ratio (95% CI)	
				Unadjusted	Adjusted^a^
Age at hydroxychloroquine initiation, y					
<45	1329	13	1.93 (1.01-3.68)	1.00 [Reference]	1.00 [Reference]
45-54	1191	29	5.20 (3.35-8.05)	2.67 (1.39-5.14)	2.48 (1.28-4.78)
55-64	1176	44	7.62 (5.29-10.91)	4.45 (2.40-8.26)	3.82 (2.05-7.14)
≥65	981	39	10.57 (6.67-16.54)	6.36 (3.39-11.94)	5.68 (2.99-10.79)
Sex					
Male	800	8	2.60 (1.11-6.02)	1.00 [Reference]	1.00 [Reference]
Female	3877	117	6.12 (4.89-7.64)	2.96 (1.45-6.06)	3.83 (1.86-7.89)
Race and ethnicity					
Asian	642	24	6.92 (4.18-11.36)	1.34 (0.85-2.12)	1.76 (1.10-2.81)
Black	491	5	2.09 (0.76-5.70)	0.35 (0.14-0.86)	0.53 (0.21-1.31)
Hispanic	828	20	5.37 (3.12-9.17)	0.84 (0.51-1.38)	1.30 (0.79-2.14)
Non-Hispanic White	2716	76	5.88 (4.46-7.75)	1.00 [Reference]	1.00 [Reference]
Indication for hydroxychloroquine					
SLE or CTD	1604	39	4.93 (3.33-7.27)	1.00 [Reference]	1.00 [Reference]
Other	383	19	11.26 (6.47-19.20)	2.16 (1.25-3.74)	1.43 (0.81-2.51)
Rheumatoid arthritis	2690	67	5.09 (3.79-6.83)	1.02 (0.68-1.51)	0.62 (0.41-0.94)
CKD stage ≥3^b^					
No	4140	98	4.99 (3.91-6.36)	1.00 [Reference]	1.00 [Reference]
Yes	537	27	10.36 (6.36-16.64)	2.56 (1.67-3.92)	1.95 (1.25-3.04)
Hydroxychloroquine dose, mg/kg^b^					
≤5	3069	32	2.09 (1.35-3.22)	1.00 [Reference]	1.00 [Reference]
5-6	653	24	7.03 (4.32-11.33)	3.45 (2.03-5.86)	3.23 (1.90- 5.50)
>6	955	69	14.01 (10.50-18.57)	6.29 (4.14-9.57)	5.30 (3.46- 8.10)
Cumulative hydroxychloroquine dose (per 100 g)^b^	NA	NA	NA	1.65 (1.45-1.87)	1.64 (1.44-1.87)
Tamoxifen use^b^					
No	4659	122	5.43 (4.36-6.76)	1.00 [Reference]	1.00 [Reference]
Yes	18	3	27.78 (7.35-75.03)	5.82 (1.85-18.29)	3.43 (1.08-10.89)
CYP2D6, CYP2C8, or CYP3A4 inhibitor use^b^					
No	3805	116	5.98 (4.74-7.52)	1.00 [Reference]	1.00 [Reference]
Yes	872	9	3.63 (1.95-6.71)	0.62 (0.37-1.04)	0.67 (0.39-1.13)
Diabetes^b^					
No	4353	119	5.53 (4.43-6.89)	1.00 [Reference]	1.00 [Reference]
Yes	324	6	5.64 (2.02-15.19)	0.80 (0.35-1.81)	0.72 (0.31-1.63)
Liver disease^b^					
No	4572	121	5.50 (4.41-6.85)	1.00 [Reference]	1.00 [Reference]
Yes	105	4	6.84 (2.14-20.75)	1.41 (0.52-3.82)	1.51 (0.56-4.11)

Abbreviations: CKD, chronic kidney disease; CTD, connective tissue disease; CYP, cytochrome P450; NA, not applicable; SLE, systemic lupus erythematosus.

^a^ Each risk factor analysis adjusted for age, sex, race and ethnicity, weight-based hydroxychloroquine dose, and cumulative dose in the first 5 years of use. The model for hydroxychloroquine dose additionally adjusted for indication of use and CKD.

^b^ Assessed in the first 5 years of hydroxychloroquine use.

**Table 3.** Risk Factors for Parafoveal vs Pericentral Pattern of Hydroxychloroquine Retinopathy^a^

Characteristic	Hazard ratio (95% CI)			
	Parafoveal		Pericentral	
	Unadjusted	Adjusted^b^	Unadjusted	Adjusted^b^
Age at hydroxychloroquine initiation, y				
<55	1.00 [Reference]	1.00 [Reference]	1.00 [Reference]	1.00 [Reference]
≥55	3.55 (2.32-5.45)	3.05 (1.98-4.71)	1.38 (0.61-3.13)	1.46 (0.63-3.38)
Sex				
Male	1.00 [Reference]	1.00 [Reference]	1.00 [Reference]	1.00 [Reference]
Female	3.94 (1.60-9.67)	5.50 (2.23-13.59)	1.35 (0.40-4.53)	1.09 (0.32-3.75)
Race and ethnicity				
Asian	0.65 (0.34-1.22)	0.81 (0.43-1.53)	13.80 (4.50-42.32)	15.02 (4.82-46.87)
Black	0.15 (0.04-0.60)	0.22 (0.05-0.88)	3.93 (0.88-17.55)	5.51 (1.22-24.97)
Hispanic	0.76 (0.45-1.28)	1.16 (0.68-1.99)	2.39 (0.53-10.66)	3.06 (0.67-13.85)
White	1.00 [Reference]	1.00 [Reference]	1.00 [Reference]	1.00 [Reference]
Indication for hydroxychloroquine				
SLE or CTD	1.00 [Reference]	1.00 [Reference]	1.00 [Reference]	1.00 [Reference]
RA	1.16 (0.74-1.82)	0.73 (0.46-1.15)	0.59 (0.25-1.43)	0.61 (0.24-1.57)
Other	2.44 (1.33-4.50)	1.74 (0.93-3.24)	1.34 (0.37-4.87)	1.42 (0.38-5.32)
CKD stage ≥3				
No	1.00 [Reference]	1.00 [Reference]	1.00 [Reference]	1.00 [Reference]
Yes	2.70 (1.70-4.30)	1.92 (1.18-3.12)	1.98 (0.67-5.82)	2.64 (0.86-8.09)
Hydroxychloroquine dose, mg/kg				
≤5	1.00 [Reference]	1.00 [Reference]	1.00 [Reference]	1.00 [Reference]
>5	4.96 (3.19-7.70)	4.27 (2.74-6.65)	6.42 (2.38-17.30)	6.23 (2.27-17.10)

Abbreviations: CKD, chronic kidney disease; CTD, connective tissue disease; RA, rheumatoid arthritis; SLE, systemic lupus erythematosus.

^a^ No users of cytochrome P450 inhibitors had pericentral retinopathy.

^b^ Each risk factor analysis adjusted for age, sex, race and ethnicity, weight-based hydroxychloroquine dose, and cumulative hydroxychloroquine dose in the first 5 years of use. The model for the risk factor of hydroxychloroquine dose additionally adjusted for indication of use and CKD.

The cumulative incidence of overall hydroxychloroquine retinopathy was numerically higher among Asian patients and lower among Black patients than among Hispanic patients or non-Hispanic White patients, but there was no significant difference in the adjusted HR for overall hydroxychloroquine retinopathy according to race or ethnicity. There were differences in the incidences of parafoveal vs pericentral retinopathy pattern by race. Asian race was associated with an increased likelihood of pericentral retinopathy compared with non-Hispanic White patients (adjusted HR, 15.02 [95% CI, 4.82-46.87]) (Table 3). Of 24 total cases of hydroxychloroquine retinopathy among 642 Asian patients, 13 (54.2%) were of the pericentral pattern compared with 4 of 76 (5.3%) total cases among 2716 non-Hispanic White patients. Of the 5 cases of hydroxychloroquine retinopathy among 491 Black patients, 3 were of the pericentral retinopathy pattern. Thus, Black race appears to be associated with an increased likelihood of pericentral retinopathy (HR, 5.51 [95% CI, 1.22-24.97]). Three of the 20 cases of hydroxychloroquine retinopathy (15.0%) among 828 Hispanic patients were of the pericentral pattern. Compared with non-Hispanic White patients, there was not a significant difference in the likelihood of pericentral retinopathy.

**Figure 1.**
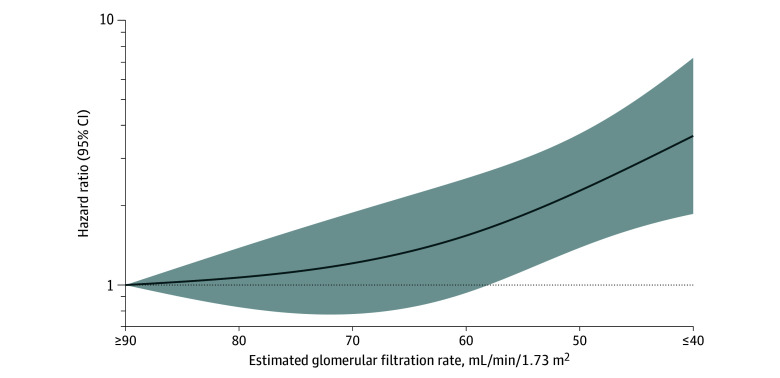
Restricted Cubic Spline Curve of Estimated Glomerular Filtration Rate and the Hazard Ratio for Hydroxychloroquine Retinopathy The curve was constructed using a multivariable Cox proportional hazards regression model, adjusting for age, sex, race and ethnicity, hydroxychloroquine weight-based dose, and cumulative hydroxychloroquine dose in first 5 years of use. Estimated glomerular filtration rate was assessed at 5 years of hydroxychloroquine use. Hydroxychloroquine retinopathy was assessed through 15 years of follow-up. The smoothed curve was fitted with restricted cubic splines with knots at 40, 60, and 90 mL/min/1.73 m^2^ or more. The reference estimated glomerular filtration rate was 90 mg/min/1.73 m^2^. The horizontal dashed line indicates a hazard ratio of 1.0. The shaded region indicates the bounds of 95% CIs for the restricted cubic spline curve.

A restricted cubic spline curve of eGFR and retinopathy HR revealed a higher risk of retinopathy with lower eGFR, particularly below 60 mL/min/1.73 m^2^ (Figure 1), corresponding with CKD stage 3 or greater. Chronic kidney disease stage 3 or greater was associated with nearly double the risk of retinopathy (adjusted HR, 1.95 [95% CI, 1.25-3.04]) (Table 2). The adjusted HR for CKD stage 3 or greater and the risk of parafoveal retinopathy was 1.92 (95% CI, 1.18-3.12) and for pericentral retinopathy was 2.64 (95% CI, 0.86-8.09) (Table 3).

There were only 3 cases of hydroxychloroquine retinopathy among 18 tamoxifen users, but there was an observed association between tamoxifen use and risk of hydroxychloroquine retinopathy (adjusted HR, 3.43 [95% CI, 1.08-10.89]) (Table 2). In contrast, CYP inhibitor medication use was not associated with retinopathy risk (adjusted HR, 0.67 [95% CI, 0.39-1.13]). Diabetes, liver disease, and indication for hydroxychloroquine use were also not associated with retinopathy risk.

**Figure 2.**
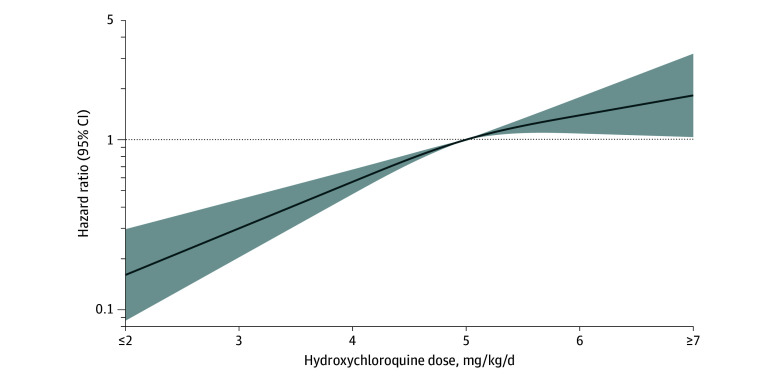
Weight-Based Hydroxychloroquine Dose and the Risk of Hydroxychloroquine Retinopathy After 15 Years of Use The curve was constructed using a multivariable Cox proportional hazards regression model, adjusting for age, sex, race and ethnicity, indication for hydroxychloroquine use, and chronic kidney disease. Hydroxychloroquine dose was assessed at 5 years of hydroxychloroquine use. Hydroxychloroquine retinopathy was assessed through 15 years of follow-up. The smoothed curve was fitted with restricted cubic splines with knots at 4, 5, and 6 mg/kg/d. The reference weight-based dose was 5 mg/kg/d. The horizontal dashed line indicates a hazard ratio of 1.0. The shaded region indicates the bounds of 95% CIs for the restricted cubic spline curve.

A higher hydroxychloroquine dose at 5 years of use was associated with an increased risk of overall hydroxychloroquine retinopathy and of retinopathy with either the parafoveal and pericentral patterns of presentation (Table 2 and Table 3). When comparing 3 dosing categories, a dose greater than 6 mg/kg/d was associated with the highest risk of hydroxychloroquine retinopathy (adjusted HR, 5.30 [95% CI, 3.46-8.10]), followed by 5 to 6 mg/kg/d (HR, 3.23 [95% CI, 1.90-5.50]) when compared with dosing of less than or equal to 5 mg/kg/d (Table 2). The restricted cubic spline curve also illustrated the association between increasing weight-based hydroxychloroquine dose and increased hydroxychloroquine retinopathy risk (Figure 2).

## Discussion

In this cohort study of long-term hydroxychloroquine users with rheumatic and dermatologic conditions, we found that increasing age, female sex, CKD stage 3 or greater, and tamoxifen use were risk factors for hydroxychloroquine retinopathy, in addition to the established risk factors of higher weight-based daily hydroxychloroquine dose and duration of use. The pericentral retinopathy pattern was observed more frequently among both Asian and Black patients than non-Hispanic White patients.

The association of hydroxychloroquine dose and duration of use with the risk of hydroxychloroquine retinopathy has been reported by prior observational studies. Many of those studies have focused on the 5 mg/kg/d cutoff for hydroxychloroquine dosing and retinopathy risk, as is currently recommended by 2016 American Academy of Ophthalmology guidelines.^4,6,7^ A previous study reported a stepwise higher incidence of hydroxychloroquine retinopathy associated with greater than 6 mg/kg dosing, followed by 5 to 6 mg/kg dosing and the lowest incidence associated with less than or equal to 5 mg/kg dosing.^5^ In the current study, we further characterized the dose-response association using a restricted cubic spline curve, and we identified a relatively linear association between hydroxychloroquine dose and the risk of retinopathy. This finding may suggest that rather than considering any particular dose to be a safe threshold (as suggested by prior prevalence studies), prescribers should use the lowest hydroxychloroquine dose that provides disease control and be wary of using higher doses for an extended period of time.

This study provides evidence for other key risk factors for hydroxychloroquine retinopathy beyond hydroxychloroquine exposure itself. After accounting for differences in weight-based dose, female sex was still associated with an approximately 4 times higher risk of incident hydroxychloroquine retinopathy compared with males. As most hydroxychloroquine users are female, this finding can also be interpreted as male sex is associated with a lower risk of retinopathy. A biological explanation for this finding is not known, but sexual disparities have been previously recognized in other retinal and ocular conditions.^19^ Older age at hydroxychloroquine initiation was strongly associated with a higher risk of hydroxychloroquine retinopathy, with approximately 3, 4, and 6 times higher risk for those aged 45 to 54 years, 55 to 64 years, and 65 years or older, respectively, compared with those younger than 45 years at time of hydroxychloroquine initiation. The retina is known to thin with age, and these age-related changes may render older individuals more vulnerable to the toxic effects of hydroxychloroquine.^20^ CKD stage 3 or greater was also found to be a risk factor for hydroxychloroquine retinopathy. Given the important role of the kidneys in clearance of hydroxychloroquine,^21^ renal insufficiency likely leads to a patient being effectively exposed to a higher systemic dose of the medication. We did not observe an association between the use of CYP inhibitors and hydroxychloroquine retinopathy risk. CYP enzymes convert hydroxychloroquine to active metabolites, which also ultimately undergo renal clearance. It is not well understood whether these metabolites are also associated with retinopathy risk.^22,23^ Future studies could replicate our findings and assess the potential association of genetic polymorphisms of CYP enzymes with retinopathy risk. Although tamoxifen use was rare in our study, we did substantiate a higher retinopathy risk among tamoxifen users. Tamoxifen itself can cause retinopathy, and there may be a synergetic toxic effect with hydroxychloroquine on the retina.^6^ Consideration of these additional risk factors should influence individualized hydroxychloroquine dosing and monitoring for hydroxychloroquine retinopathy.

We confirmed the previously reported association of pericentral retinopathy with Asian race,^10,13^ and our findings suggest a possible association with the pericentral retinopathy pattern among Black patients. As there were relatively few cases of hydroxychloroquine retinopathy identified among Black patients in this cohort, this finding awaits replication in other settings. We cannot offer a pathophysiological explanation for racial differences in the pericentral vs parafoveal retinopathy patterns. Genetic risk factors for hydroxychloroquine retinopathy have been proposed but not yet tested on a large scale, and no associations have been confirmed.^24^ Racial and ethnic disparities in hydroxychloroquine adherence and in the receipt of guideline-recommended care have been previously observed among Black patients with SLE.^25,26,27^ The pericentral pattern is not considered a more advanced form of hydroxychloroquine retinopathy than the parafoveal pattern, but it may be identified at a later stage because eye care clinicians may be less familiar with its appearance. Further work is needed to understand the observed racial and ethnic differences in retinopathy risk and pattern. In addition, age, sex, and CKD were associated with the parafoveal pattern, but we did not find significant associations with the pericentral retinopathy pattern. The relatively small number of pericentral retinopathy cases likely limited those analyses. Therefore, studies with a larger number of pericentral retinopathy cases would be needed to assess whether these risk factors for overall hydroxychloroquine retinopathy are also associated with the pericentral pattern. In the meantime, awareness of the pericentral pattern is important to screen properly for hydroxychloroquine retinopathy in all patients.

### Strengths and Limitations

This study has some strengths, including the use of a cohort with representation from multiple racial and ethnic groups and long-term follow-up. Hydroxychloroquine exposure was assessed from comprehensive pharmacy dispensing records, which account for adherence by ensuring medication possession and therefore represent a more accurate estimation of exposure than prescribed or reported dose. Outcomes were validated on masked readings of guideline-recommended spectral domain–optical coherence tomography retinopathy scans by expert ophthalmologists, whereas prior studies have relied on physician report or patient self-report of retinopathy or on less sensitive screening modalities; therefore, this study should be less susceptible to misclassification bias.

Our study has several limitations worth noting. We lacked data on hydroxychloroquine blood levels, so we could not test the potential association with hydroxychloroquine retinopathy risk, which is a topic of current debate.^9,18^ As hydroxychloroquine retinopathy is typically asymptomatic until the late stage, early diagnosis must be based on active screening. Therefore, the date of onset could have been earlier than recognized for some patients. Although this is the largest cohort study to our knowledge of risk factors for incident hydroxychloroquine retinopathy to date, there were still small numbers of events within some of the risk categories, and there were a limited number of patients with pericentral retinopathy.

## Conclusions

In this cohort study, we identified several key risk factors for incident hydroxychloroquine retinopathy in addition to hydroxychloroquine dose. These findings are relevant to long-term users of this important and commonly prescribed medication for patients with SLE and other rheumatic and dermatologic conditions. Female sex, older age, CKD stage 3 or greater, and tamoxifen use were each associated with an increased risk of hydroxychloroquine retinopathy, whereas male sex, younger age, and normal kidney function were associated with a lower risk of hydroxychloroquine retinopathy. These factors should influence hydroxychloroquine dosing and monitoring for this complication. Asian and Black patients were observed to have a higher incidence of the pericentral retinopathy pattern. Because recognition of the pericentral pattern requires wider examination of the fundus than standard optical coherence tomography recordings or central 10-2 visual fields provide, specialists who interpret hydroxychloroquine retinopathy screening studies must be aware of this pattern to ensure timely detection. Personalized evaluation of these risk factors for hydroxychloroquine retinopathy should guide the optimal use of this medication.

## Notes

Supplement 1. **eTable.** Common CYP Inhibitors of Hydroxychloroquine Metabolism

Supplement 2.
Data Sharing Statement
