# Supplementary material for: Risk Factors for Hydroxychloroquine Retinopathy and Its Subtypes
Source: JAMA Netw Open. 2024 May 9;7(5):e2410677. doi: 10.1001/jamanetworkopen.2024.10677 (PMC11082687; doi:10.1001/jamanetworkopen.2024.10677)
Supplement: Supplement 1. — eTable. Common CYP Inhibitors of Hydroxychloroquine Metabolism [file jamanetwopen-e2410677-s001.pdf]

Supplementary Online Content

Jorge AM, Melles RB, Marmor MF, Zhou B, Zhang Y, Choi HK. Risk factors for hydroxychloroquine retinopathy and its subtypes. *JAMA Netw Open.* 2024;7(5):e2410677. doi:10.1001/jamanetworkopen.2024.10677

eTable. Common CYP Inhibitors of Hydroxychloroquine Metabolism

This supplementary material has been provided by the authors to give readers additional information about their work.

© 2024 Jorge AM et al. *JAMA Network Open.*

eTable. Common CYP Inhibitors of Hydroxychloroquine Metabolism

**CYP2D6 inhibitors**	Strong inhibitors: Fluoxetine, bupropion, paroxetine<br>Weak inhibitors: amiodarone, celecoxib, cimetidine, sertraline, labetalol, escitalopram
**CYP2C8 inhibitors**	Montelukast, gemfibrozil, clopidogrel, salmeterol, felodipine
**CYP3A4 inhibitors**	Ketoconazole, clarithromycin, diltiazem, lopinavir (and other HIV protease inhibitors), tamoxifen, verapamil

© 2024 Jorge AM et al. *JAMA Network Open.*
