# Supplementary material for: Risk Factors for Hydroxychloroquine Retinopathy and Its Subtypes
Source: JAMA Netw Open. 2024 May 9;7(5):e2410677. doi: 10.1001/jamanetworkopen.2024.10677 (PMC11082687; doi:10.1001/jamanetworkopen.2024.10677)
Supplement: Supplement 2. — Data Sharing Statement [file jamanetwopen-e2410677-s002.pdf]

Data Sharing Statement

Jorge. Risk Factors for Hydroxychloroquine Retinopathy and Its Subtypes. *JAMA Netw Open*. Published May 09, 2024. doi:10.1001/jamanetworkopen.2024.10677

Data
Data available: No

Additional Information
Explanation for why data not available: The data that support the findings of this study are available from Kaiser Permanente Northern California. Restrictions apply to the availability of these data, which were used under a data use agreement for this study. Data are available from the authors with the permission of Kaiser Permanente Northern California.
